# Low-intensity pulsed ultrasound activates the phosphatidylinositol 3 kinase/Akt pathway and stimulates the growth of chondrocytes in three-dimensional cultures: a basic science study

**DOI:** 10.1186/ar2451

**Published:** 2008-07-11

**Authors:** Ryohei Takeuchi, Akihide Ryo, Noriko Komitsu, Yuko Mikuni-Takagaki, Atsuko Fukui, Yuta Takagi, Toshihiko Shiraishi, Shin Morishita, Yoshiyuki Yamazaki, Ken Kumagai, Ichiro Aoki, Tomoyuki Saito

**Affiliations:** 1 Department of Orthopaedic Surgery, Yokohama City University School of Medicine, 3-9 Fukuura, Kanazawa-ku, Yokohama City, Kanagawa 236-0004, Japan; 2 First Research Group, AIDS Research Center, National Institute of Infectious Diseases, 4-7-1 Gagkuen, Musashimurayama, Tokyo 208-0011, Japan; 3 Department of Pathology, Yokohama City University School of Medicine, 3-9 Fukuura, Kanazawa-ku, Yokohama City, Kanagawa 236-0004, Japan; 4 Department of Functional Biology, Kanagawa Dental College, 82 Inaokachyo, Yokosuka City, Kanagawa 238-8580, Japan; 5 Department of Environment and Information Sciences, Yokohama National University Graduate School, 79-5 Tokiwadai, Hodogaya-ku, Yokohama City, Kanagawa 240-851, Japan

## Abstract

**Introduction:**

The effect of low-intensity pulsed ultrasound (LIPUS) on cell growth was examined in three-dimensional-cultured chondrocytes with a collagen sponge. To elucidate the mechanisms underlying the mechanical activation of chondrocytes, intracellular signaling pathways through the Ras/mitogen-activated protein kinase (MAPK) and the integrin/phosphatidylinositol 3 kinase (PI3K)/Akt pathways as well as proteins involved in proliferation of chondrocytes were examined in LIPUS-treated chondrocytes.

**Methods:**

Articular cartilage tissue was obtained from the metatarso-phalangeal joints of freshly sacrificed pigs. Isolated chondrocytes mixed with collagen gel and culture medium composites were added to type-I collagen honeycomb sponges. Experimental cells were cultured with daily 20-minute exposures to LIPUS. The chondrocytes proliferated and a collagenous matrix was formed on the surface of the sponge. Cell counting, histological examinations, immunohistochemical analyses and western blotting analysis were performed.

**Results:**

The rate of chondrocyte proliferation was slightly but significantly higher in the LIPUS group in comparison with the control group during the 2-week culture period. Western blot analysis showed intense staining of type-IX collagen, cyclin B_1 _and cyclin D_1_, phosphorylated focal adhesion kinase, and phosphorylated Akt in the LIPUS group in comparison with the control group. No differences were detected, however, in the MAPK, phosphorylated MAPK and type-II collagen levels.

**Conclusion:**

LIPUS promoted the proliferation of cultured chondrocytes and the production of type-IX collagen in a three-dimensional culture using a collagen sponge. In addition, the anabolic LIPUS signal transduction to the nucleus via the integrin/phosphatidylinositol 3-OH kinase/Akt pathway rather than the integrin/MAPK pathway was generally associated with cell proliferation.

## Introduction

The degenerative abrasion of cartilage tissue due to aging and a malalignment of the lower extremities causes osteoarthritis. Moreover, articular cartilage is a tissue that is difficult to regenerate once damaged. Many attempts have therefore been made to achieve regeneration of damaged cartilage tissue. Conservative treatments include physiotherapy, such as quadriceps muscle training, or the intra-articular injection of hyaluronic acid. The regeneration of normal cartilage tissue, however, has not yet been achieved [1]. The elements that promote the regeneration of cartilage include growth factors [2], soluble mediators [3], corrections of any malalignment and mechanical stimulation [4-6].

Surgical treatments include a high tibial osteotomy, the micro-fracture method, transplantation of osteocartilaginous plugs [7], and transplantation of cultured cartilage [8]. During the transplantation of cultured cartilage, a key part of the procedure is the *in vitro *preparation of high-quality cartilage tissue prior to transplantation [9]. Mechanical stimulation is one of the essential factors that promotes the differentiation and proliferation of intact chondrocytes as well as *in vitro *cultures for transplantation. Various methods of mechanical stimulation of chondrocytes have been reported, such as loading with hydrostatic pressure [10], the application of tensile stress against the culture scaffold [11], oscillation using a vibrator [12] and low-intensity pulsed ultrasound (LIPUS) [13-15].

The matrix surrounding the chondrocytes also plays an important role in the proliferation and survival of chondrocytes. Through this extracellular matrix, chondrocytes receive various kinds of extracellular information such as mechanical signals and hormonal mediators. Mechanical stimulation has been reported to activate chondrocytes and to promote their synthesis of the extracellular matrix. Few reports have focused on the signal transmission, however, which results in chondrocyte activation. To characterize these mechano-transduction pathways in chondrocytes, we have previously established a new three-dimensional (3D) culture system, which forms a tissue architecture similar to the structure of articular cartilage tissue *in vivo *[12]. The effects of vibration on chondrocytes were previously examined in this system, and the involvement of a mechano-transduction pathway via the integrin/mitogen-activated protein kinase (MAPK) pathway and of another signaling pathway via β-catenin was evaluated. Although many previous studies reported that osteoblasts are activated by LIPUS, which has been widely used in clinical settings to accelerate the process of fracture healing, its practical use for cartilage repair in a clinical setting is so far limited [16-18].

The present study demonstrates that the combination of the 3D chondrocyte culturing technique with LIPUS not only promotes the production of type-IX collagen, but also significantly increases the number of chondrocytes. In addition, the results indicate the potential involvement of the integrin/phosphatidylinositol 3-OH kinase (PI3K)/Akt pathway downstream of LIPUS exposure, rather than the integrin/MAPK/MAPK kinase pathway, which is generally involved in the induction of cellular proliferation.

## Materials and methods

### Cell cultures

Articular cartilage tissue was obtained from the metatarso-phalangeal joints of freshly slaughtered 6-month-old pigs in a slaughterhouse. Articular cartilage slices were cut into smaller pieces, and the cartilage specimens were washed well in PBS (pH 7.4) and digested with 0.25% trypsin–ethylenediamine tetraacetic acid (Gibco, Grand Island, NY, USA) for 20 minutes. The resultant chondrocyte preparations were washed again with PBS to remove the trypsin, and were then incubated for about 8 hours in Dulbecco's modified Eagle's medium (DMEM; Gibco) supplemented with 0.1% type-II collagenase (Worthington Biochemical Co., Lakewood, OH, USA), 10% heat-inactivated FBS (Equitech-Bio, Inc., Kerrville, TX, USA) and antibiotics. The chondrocytes were subsequently isolated and washed with culture medium, collected by centrifugation (2,000 rpm, 37°C, 5 min), and then mixed with 0.2% atelocollagen gel (type-I collagen derived from bovine tendons; Koken Co., Tokyo, Japan) containing culture medium (DMEM; Gibco).

Twenty-four-well plates containing type-I honeycomb collagen sponges (discs with a diameter of 15 mm and thickness of 2 mm; Koken Co.) at the bottom of each well were used as 3D carriers of the chondrocyte culture [19]. Chondrocytes in the atelocollagen gel and also chondrocytes in the culture medium composites were added to each sponge and were incubated at 37°C for 1 hour. The final cell density was adjusted to 2 × 10^6 ^cells/well/ml [12]. After the collagen sponge and cell–collagen gel composites became stiff, they were then incubated with 2 ml complete DMEM in 5% CO_2_/95% air at 37°C, and the cultured medium was replaced with fresh DMEM containing L-ascorbic acid (50 μg/ml) twice weekly.

### Low-intensity pulsed ultrasound stimulation

The sonic accelerated fracture healing system (Exogen Inc., Piscataway, NJ, USA), a LIPUS apparatus, was used to deliver an ultrasound signal. The sonic accelerated fracture healing system is one of the instruments in current clinical use in cases of delayed repair of a fracture. The temporal average intensity was 30 mW/cm^2 ^and the frequency was 1.5 MHz with a 200-μs tone burst repeated at 1.0 KHz. LIPUS was applied to the chondrocytes after 24 hours in culture through the bottom of the culture dish (24-well plate) via a coupling gel and silicon rubber that had been placed between the LIPUS transducer and the dish. LIPUS was administered for 20 minutes every day in a span of this experiment. Control samples were prepared in the same manner without LIPUS. Thereafter, the cultured tissues and their supernatant medium were harvested at days 3, 7, 10 and 14.

### Cell counting

The cartilage tissues were harvested 1, 3, 7, 10 and 14 days after culture (2 hours after the last LIPUS) and were cut into smaller pieces. Each sample was then incubated for about 8 hours in DMEM (Gibco) supplemented with 0.1% type-II collagenase (Worthington Biochemical Co.), 10%-heat-inactivated FBS (Equitech-Bio, Inc.) and antibiotics. The chondrocytes were then isolated, washed with culture medium, and collected by centrifugation (2,000 rpm, 37°C, 5 min). After the supernatant medium was removed, a solution containing 0.1 M. citric acid and 0.1% crystal violet was added to the cells and then the cells were counted using a hemocytometer (Burker-Turk, Tokyo, Japan).

### Histological examinations

Histological evaluations of the specimens were conducted at weeks 1 and 2 post culture. The specimens were fixed overnight in 4% paraformaldehyde in PBS, paraffin-embedded, sectioned to a 5 μm thickness, and were stained with Alcian blue and Safranin O. For each sample, at least two different section levels and two histological sections for each level were analyzed. The sections were analyzed and photographed using an Olympus photomicroscope BX-50 (Olympus Co., Tokyo, Japan).

### Immunohistochemistry

Immunohistochemical analyses were conducted with antibodies raised against anti-type-II collagen antibody (1:100; Fuji Pharm. Lab., Toyama, Japan) and against anti-type-IX collagen (1:100; Chemicon International, Billerica, MA, USA) using week 1 and week 2 postcultures to evaluate the expression of the chondrocyte phenotype and also to assess the type-II and type-IX collagen production levels. The specimens from the 1-week and 2-week postcultures that were harvested 2 hours after the last LIPUS were fixed in 4% paraformaldehyde in 0.1 M PBS (pH 7.4), and 16-μm cryostat sections were made.

For further confirmation of chondrocyte growth, Ki67 staining was performed because this factor has been shown to be a very reliable proliferation marker [20]. The monoclonal mouse anti-human antibody Ki67 (MIB1; DAKO, Glostrup, Denmark), which also shows cross-reactivity with porcine tissues, was used to determine the extent of proliferation. The sections cultured at day 7 were incubated with this Ki67 primary antibody, followed by a secondary biotinylated anti-rabbit antibody and horseradish peroxidase–avidin complex (DAKO). The color reaction was developed by 3,3'-diaminobenzidine substrate, followed by counterstaining with hemalaun (Merck, Frankfult, Germany). Chondrocytes showing a definite nuclear staining pattern were scored as positive. All slides were reviewed by two investigators without any prior knowledge of the experiment. Five different randomly chosen areas were reviewed in five different specimens, and the number of Ki67-positive cells per 100 chondrocytes was counted in each slice. The percentages of positive cells (MIB1 index) were then calculated.

Quantitative evaluations were conducted using specimens stained with an anti-β-catenin antibody (Acris, Herford, Germany). The nuclear translocation of β-catenin was visible by brown staining. After counting 100 cells, the ratio of the cells whose nuclei were stained brown was compared between the ultrasound group and the control group. All slides were reviewed by two investigators without any prior knowledge of the experiment. In five different randomly chosen areas in five different specimens, the number of β-catenin antibody-positive stained cells per 100 chondrocytes was counted in each slice. The percentages of positive cells were then calculated.

### Western blotting analysis

For the western blotting analysis of the specimens cultured for 1 week, cartilage tissues specimens were harvested 2 hours after the last LIPUS and were cut into smaller pieces. Each sample was then incubated for about 8 hours in DMEM (Gibco) supplemented with 0.1% type-II collagenase (Worthington Biochemical Co.), 10%-heat-inactivated FBS (Equitech-Bio, Inc.) and antibiotics. The chondrocytes were then isolated, washed with culture medium, and collected by centrifugation (2,000 rpm, 37°C, 5 min). After the supernatant medium was removed, the cells were rinsed with 200 μl PBS, filtered by centrifugation, and added to a 200 μl aliquot of 2× sample buffer (62.5 mmol/l Tris–HCl (pH 6.8), 2% SDS, 10% glycerol, 50 mmol/l dithiothreitol, 0.01% bromophenol blue). The cell lysates were then boiled for 10 minutes at 75°C.

Equal amount of the proteins were separated on a 10% SDS–polyacrylamide gel at 200 V, 25 mA for 80 minutes and were transblotted to nitrocellulose membranes (Millipore, Billerica, MA, USA) using a wet transfer system (BIO-RAD, Hercules, CA, USA) at 200 V, 150 mA for 60 minutes. The membranes were blocked with blocking buffer (5% skimmed milk in TBS and 0.05% Tween 20 and Blocking One–P; Nacalai Tesque Inc., Kyoto, Japan) and were incubated with the following antibodies: anti-Akt (Rockland, Gilbertsville, PA, USA), anti-phospho-Akt (Cell Signaling Technology, Beverly, MA, USA), anti-MAPK and anti-phospho-MAPK (Cell Signaling Technology), anti-cyclin D_1 _(Biosource, Camarillo, CA, USA), anti-cyclin B_1 _and anti-focal adhesion kinase (anti-FAK; Upstate Cell Signaling Solutions, NY, USA), anti-phospho-FAK (Rockland), anti-collagen-II (Chemicon International), and anti-collagen-IX (Cell Signaling Technology).

After incubation with the corresponding horseradish peroxidase-conjugated secondary antibodies (dilution: 1/5,000), membranes were finally incubated with a chemiluminescent reagent (NEL103; Perkin Elmer Life Science, Fremont, CA, USA) and the signals produced were recorded on X-ray film (BIOMAX XAR Film, Rochester, Minesota, USA) for a densitometric analysis. The effects of PI3K inhibitor (LY294002; Cell Signaling Technology) and MEK1 inhibitor (PD98059; Cell Signaling Technology) for cell growth were studied. Chondrocytes were pretreated with MEK1 inhibitor (250 μM/ml) and PI3K inhibitor (250 μM/ml) for 12 hours and 24 hours, followed by stimulation with LIPUS for 20 minutes. Each sample was harvested 2 hours after LIPUS stimulation and the influence of these inhibitors was judged by western blotting analysis of proliferating cell nuclear antigen (PCNA; DAKO).

### Statistical analysis

Data are expressed as the mean ± standard deviation. Quantitative evaluations of Ki67-positive cells and β-catenin-positive cells were assessed by Mann–Whitney's U test. The change in the number of chondrocytes was assessed using repeated-measures analysis of variance. *P *< 0.05 was considered significant.

## Results

### Histological specimens

**Figure 1:**
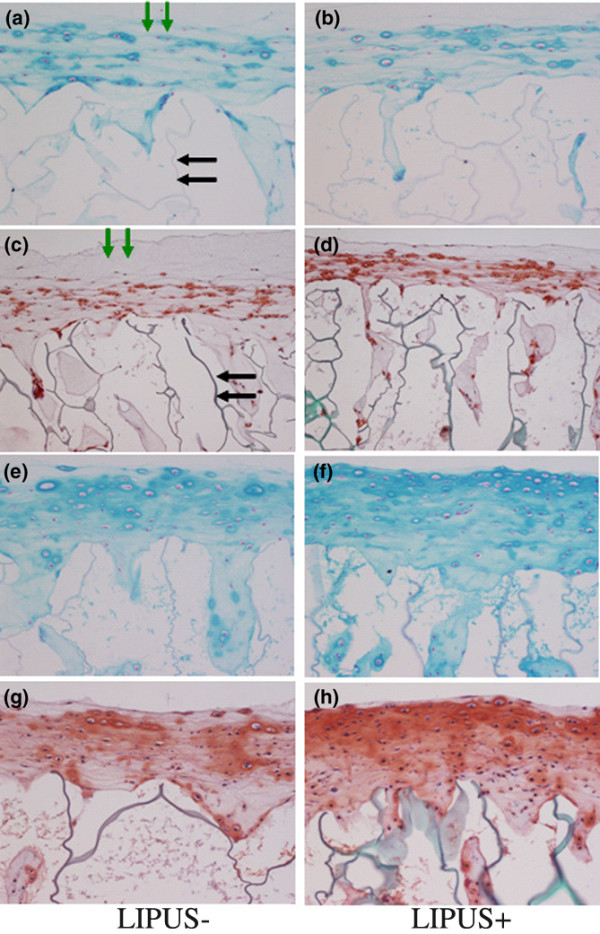
**High-magnification sections of chondrocyte–collagen sponges 1 week after culture**. Cartilage layers with a laminar structure on the collagen sponges (green arrows) and a grey structure, which represents the walls of collagen sponges (black arrows), are visible at high magnification (100×). **(a) **to **(d) **Specimens at week 1 of culture. (a) and (b) Alcian blue staining, and (c) and (d) Safranin O staining: many chondrocytes with blue-stained and red-stained peripheral matrices could be observed, respectively. The chondrocytes exhibited a layer structure, and their infiltration into the sponge can also be observed. **(e) **to **(h) **Specimens at week 2 of culture. (e) and (f) Alcian blue staining, and (g) and (h) Safranin O staining: many chondrocytes with blue-stained and red-stained peripheral matrices can be observed, respectively. The layer of chondrocytes that formed on the surface of the sponge was found to be thicker in comparison with the week 1 cultures, and the volume of the extracellular matrix had also increased. The cartilage tissue that formed on the surface of the sponge consisted of more than 10 layers of chondrocytes. The staining of the extracellular matrix in the LIPUS group was also found to be stronger than in the control group.

After 1 week of culture, cartilaginous tissue consisting of at least five cell layers was formed on the collagen sponges in both the control group and the LIPUS group (Figure 1a to 1d). Simultaneously, both the penetration of chondrocytes and the formation of an extracellular cartilage matrix were observed inside the collagen sponge. In addition, an extracellular matrix rich in proteoglycans and intensively stained with Alcian blue and Safranin O was observed surrounding the chondrocytes.

During week 2 of culture in the 3D system, the cartilaginous tissue in each specimen appeared thicker in comparison with week 1, and the volume of the extracellular matrix had also increased and formed a stable cartilaginous tissue. The thickness of the tissue in week 2 was found to be greater in the LIPUS group than in the control group, and the staining of the matrix, especially near the surface, was also more intense in the LIPUS group (Figure 1e to 1h). The ratio between the number of cells in the cartilage layer that had formed on the collagen sponge and in the sponge was approximately 2:1.

### Growth curves of the chondrocytes

**Figure 2:**
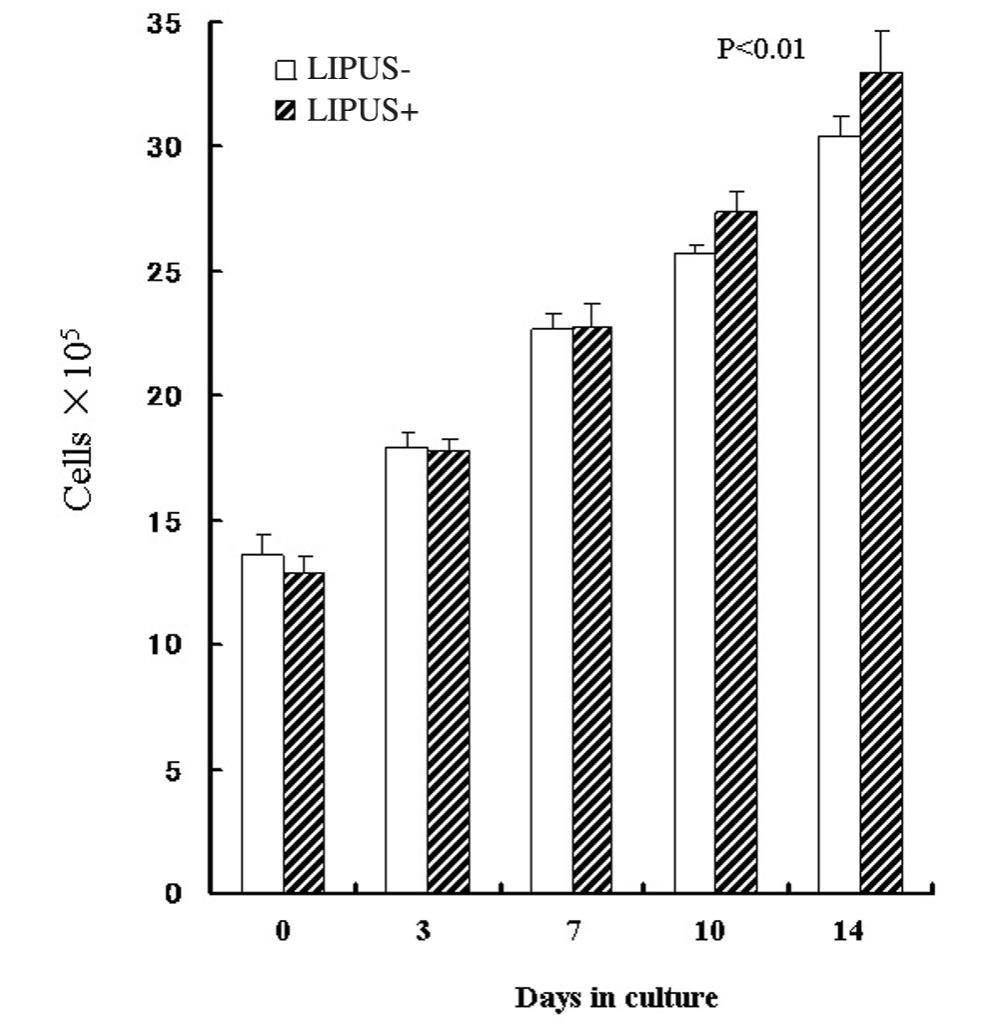
**Growth curves of the cells in the chondrocyte–collagen sponges (n = 7)**. A time-dependent increase in the number of chondrocytes can be seen in both the low-intensity pulsed ultrasound (LIPUS) group (US+) and in the control group (US-). The rate of increase in the chondrocytes number was significantly greater, however, in the LIPUS group in comparison with the control group (*P *< 0.01). The change in the number of chondrocytes was assessed using repeated-measures analysis of variance.

The initial results demonstrated that LIPUS facilitates the formation of a 3D structure of cartilage tissue, suggesting that increased cell proliferation had occurred. The effect of LIPUS on cell proliferation was therefore examined in the culture system. The number of live chondrocytes on day 0 was (13.6 ± 0.8) × 10^5 ^and (12.9 ± 0.6) × 10^5 ^cells in the control and LIPUS groups, respectively. A time-dependent increase in the total number of chondrocytes was noted, and on day 14 the cell counts were (30.4 ± 0.8) × 10^5 ^and (33.0 ± 1.7) × 10^5 ^in the control group and the LIPUS group, respectively. There was therefore a small but significantly greater increase in the cell number observed in the LIPUS group in comparison with the control group (*P *< 0.01; Figure 2).

### Type-II collagen and type-IX collagen

Collagen is essential for the formation of cartilage tissue and also for the proliferation of chondrocytes. Furthermore, the current results demonstrated the formation of a thicker cartilaginous structure following LIPUS – suggesting that the increased secretion of extracellular matrix components such as the collagens had occurred.

**Figure 3:**
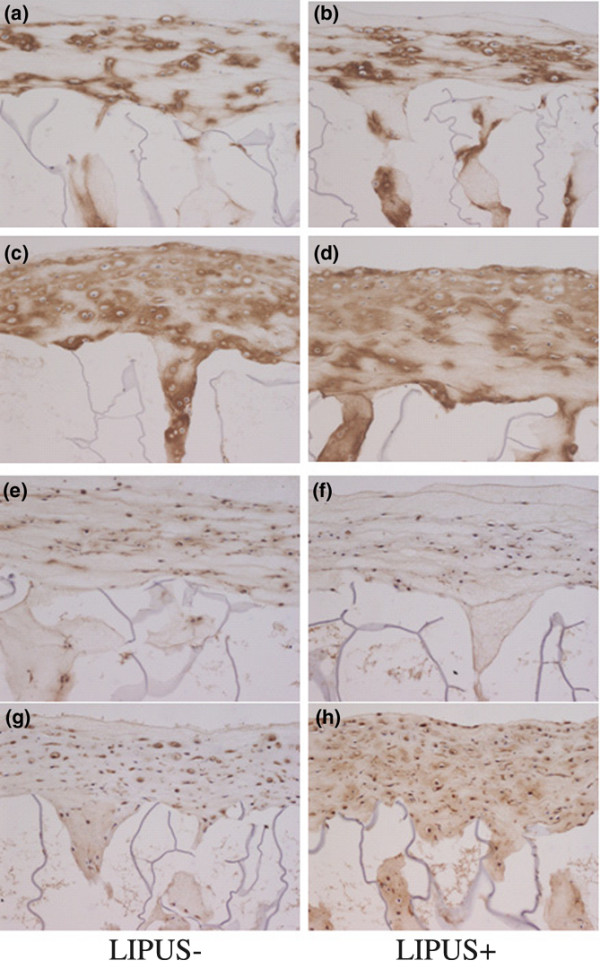
**High-magnification sections of chondrocyte–collagen sponges 1 and 2 weeks post culture**. Sections of chondrocyte–collagen sponges 1 and 2 weeks post culture at high magnification (anti-collagen antibody type-II and type-IX stain, 100×). **(a)**, **(b) **Anti-type-II collagen antibody staining of specimens after week 1 of culture. Brown staining of the matrix with anti-collagen type-II antibodies can be observed around the chondrocytes, indicating production of this collagen. **(c)**, **(d) **Anti-type-II collagen antibody-stained specimens after week 2 of culture. Strong brown staining of the matrix can be observed around the cells at a similar level in both groups. **(e)**, **(f) **Anti-type-IX collagen antibody-stained specimens after week 1 of culture. Positive brown staining of the matrix with anti-type-IX collagen antibodies can be observed around the cells, thus indicating the production of this collagen around the chondrocytes. **(g)**, **(h) **Anti-type-IX collagen antibody stained specimens after week 2 of culture. Positive brown staining of the matrix with anti-type-IX collagen antibodies can be observed around the cells, thus indicating production of this collagen around the chondrocytes.

Following type-II collagen antibody staining, both the chondrocyte layers, which formed cartilaginous tissue on the collagen sponge, and the matrix formed inside the sponge were strongly positive in both the LIPUS group and the control group. There were also no apparent differences in the intensities of this staining between these two groups (Figure 3a to 3d).

Type-IX collagen antibody staining of the culture specimens showed the intensity of this staining in the chondrocyte layers on the sponge to be far stronger in the LIPUS group than in the control group after 2 weeks of culture, thus indicating an accumulation of type-IX collagen (Figure 3e to 3h).

### Ki67 and β-catenin

**Figure 4:**
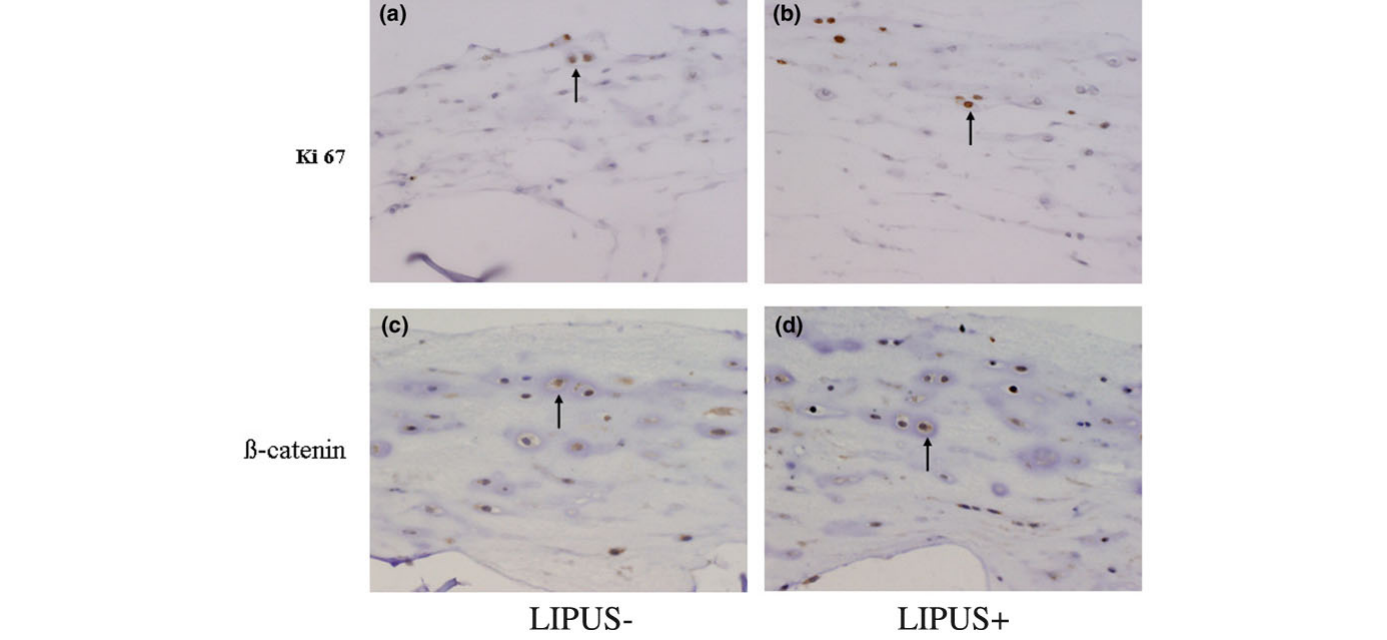
**Ki67 and β-catenin antibody staining**. **(a)**, **(b) **Anti-Ki67 antibody staining of week 2 cultures (200× magnification). The nuclei are positively stained with an anti-Ki67 antibody in both the control group (US-) and the low-intensity pulsed ultrasound (LIPUS) group (US+) (black arrows). **(c)**, **(d) **Anti-β-catenin antibody staining of week 2 cultures (200× magnification). The nuclei are positively stained with an anti-β-catenin antibody in both the control group (US-) and the LIPUS group (US+) (black arrows).

Immunohistochemical staining with Ki67 revealed distinctive labeling in the chondrocyte nuclei (Figure 4a,b). The cells with brown-stained nuclei were considered Ki67-positive. The large number of Ki67-positive cells indicated that LIPUS stimulated cell proliferation. In the cells in which β-catenin had translocated to the nucleus, brown nuclear staining with an anti-β-catenin antibody was evident (Figure 4c,d).

### Quantitative evaluation of both Ki67-positive cells and β-catenin-positive cells

**Figure 5:**
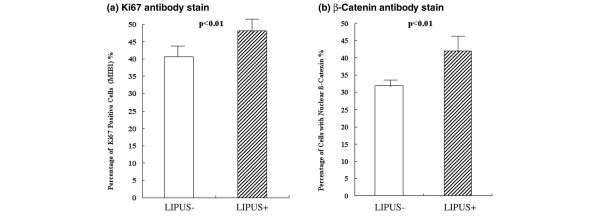
**Quantitative evaluation of Ki67-positive cells and β-catenin-positive cells**. After counting 100 cells in each specimen in the low-intensity pulsed ultrasound (LIPUS) group (US+) and the control group (US-), the numbers of cells with positively stained nuclei were compared for both **(a) **Ki67 and **(b) **β-catenin. There were significantly more brown stained cells in the LIPUS group in both cases (*P *< 0.01).

The Ki67 index of the chondrocytes exposed to LIPUS was found to be 48 ± 3.7%, in comparison with 41 ± 3.0% in the control group (Figure 5a), which was significantly different. The average percentage of β-catenin-positive cells with brown-stained nuclei (that is, positive cells) was determined to be 42 ± 4.9% in the LIPUS group and 32 ± 2.7% in the control group. This indicated a significant difference between the two groups (Figure 5b).

### Western blotting analysis

#### Collagen type-II

**Figure 6:**
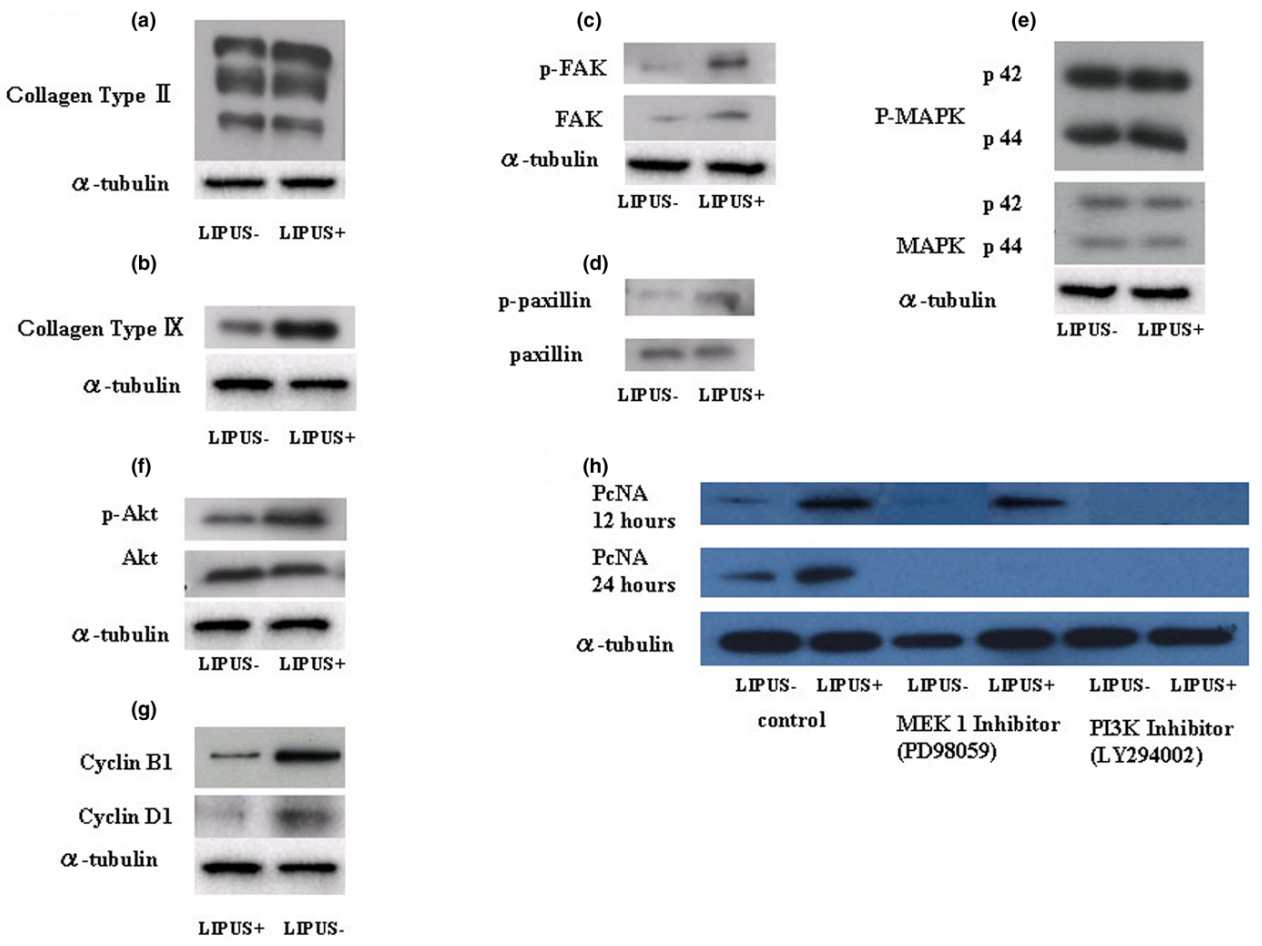
**Western blotting analysis**. **(a) **Type-II collagen. **(b) **Type-IX collagen. **(c) **Focal adhesion kinase (FAK) and phosphorylated FAK (p-FAK). **(d) **Paxillin and phosphorylated Paxillin (p-Paxillin). **(e) **Mitogen-activated protein kinase (MAPK) and phosphorylated MAPK (p-MAPK). There are no evident differences in the expression levels of total MAPK and p-MAPK between the two groups. **(f) **Akt and phosphorylated Akt (p-Akt). There were no differences found in the intensity the total Akt expression between the two groups, but p-Akt was found at higher levels in the LIPUS group (US+) in comparison with the control group (US-). **(g) **Cyclin B_1 _and cyclin D_1_. **(h) **Changes of proliferating cell nuclear antigen (PCNA) using MEK1 inhibitor (PD98059) and phosphatidylinositol 3-OH kinase (PI3K) inhibitor (LY294002). Chondrocytes were pretreated with MEK1 inhibitor (PD98059, 250 μM/ml) and PI3K inhibitor (LY294002, 250 μM/ml) for 12 hours and 24 hours followed by stimulation with LIPUS for 20 minutes. Each sample was harvested 2 hours after LIPUS stimulation and the influence of these inhibitors was judged in western blotting analysis of the expression of PCNA.

A western blot analysis showed immunoreactive bands for collagen type-II were observed at about 200 kDa, and were found to be present at a similar intensity in the LIPUS group and the control group (Figure 6a).

#### Collagen type-IX

An immunoreactive western band for collagen type-IX of about 110 kDa was detected at a higher level in the LIPUS group in comparison with the control group (Figure 6b).

#### FAK, phosphorylated FAK, Paxillin and phosphorylated Paxillin

Immunoreactive bands corresponding to FAK and phosphorylated FAK were detected by western blotting at about 125 kDa (Figure 6c). Positive bands for Paxillin and its phosphorylated form were observed at about 68 kDa (Figure 6d). Although the levels of total FAK and Paxillin were similar with and without LIPUS exposure, the staining of their phosphorylated counterparts was stronger in the LIPUS group than in the control group (Figure 6c,d). These data thus indicate that LIPUS exposure results in the activation of both FAK and Paxillin.

#### MAPK and phosphorylated MAPK

Whereas MAPK and phosphorylated MAPK (p-42, p-44) were both detected in both the LIPUS group and the control group, there were no evident differences in the intensity (Figure 6e).

#### Akt and phosphorylated Akt

Akt, a cell survival signal, was found to be similarly expressed in both the LIPUS group and the control group but was observed to be phosphorylated to a greater extent in the LIPUS group (Figure 6f). These results indicate that LIPUS increased cell proliferation in this culture system by preferentially activating the PI3K/Akt pathway rather than the MEK/MAPK pathway.

#### Cyclin B_1 _and cyclin D_1_

Consistent with the increased chondrocyte growth, the expression of the cell proliferation markers cyclin D_1 _and cyclin B_1 _was enhanced in both cases by LIPUS. The expression of both of these cyclins was also detected at higher levels in the LIPUS group in comparison with the control group (Figure 6g). These results confirm that the increase in cell numbers in response to LIPUS coincide with the enhanced expression of these two cyclins.

#### Changes of proliferating cell nuclear antigen using MEK1 inhibitor and PI3K inhibitor

The influence of the MEK1 inhibitor (PD98059) and of the PI3K inhibitor (LY294002) was judged in western blotting analysis of PCNA. The expression of PCNA at 12 hours was decreased by PD98059 in the LIPUS-negative group and was detected at higher level in the LIPUS-positive group in comparison with the LIPUS-negative group, but the expression of PCNA at 24 hour was completely decreased by this inhibitor in both the LIPUS-negative and LIPUS-positive groups. Cell growth according to LIPUS is hypothesized to depend not only on a MAPK cascade but also on the effect of other signal transductions. The expression of PCNA at 12 and 24 hours, however, was completely decreased by PI3K inhibitor (LY294002).

## Discussion

### LIPUS promotes proliferation of chondrocytes

Previous studies indicated that LIPUS increases the production of the extracellular matrix around chondrocytes, but not the actual proliferation of the chondrocytes themselves. Zhang and colleagues have reported that although pulsed low-intensity ultrasound increases the number of hypertrophic chondrocytes around the callus of healing fractures, it does not alter the hyaline cartilage [15]. Nishikori and coworkers have also reported that chondrocytes can be grown in a 3D collagen gel without loss of their chondrogenic phenotype but that LIPUS did not enhance cell proliferation in either a monolayer culture or a 3D culture [13]. In this same study, ultrasound exposure was found to be advantageous in inducing chondrocyte production of collagen gel composites with mature aggrecan.

Parvizi and colleagues irradiated the rat monolayer culture cells at 1 MHz to investigate the [^3^H]thymidine incorporation levels, the DNA contents, the mRNA levels of α(I) and α(II) procollagens and the mRNA contents of proteoglycans inducing aggrecan. The group reported that the irradiation increased the aggrecan mRNA and proteoglycan levels without any significant effects upon the proliferation of chondrocytes [14].

A number of studies have reported a slight increase in the number of chondrocytes following the use of the same therapeutic low-intensity pulsed ultrasound, which may be called PLIUS. Zhang and colleagues previously irradiated cultured chondrocytes at 2 mW/cm^2 ^and 30 mW/cm^2^, and measured the cell count and volume of the extracellular matrix over time. At 2 mW/cm^2^, they reported that the extracellular matrix as well as the cell number increases significantly but only transiently on day 3 of culture, in comparison with the control group [15]. In the current study with a 3D culture system, the number of chondrocytes doubled by the end of the 2-week incubation in both groups. This rate of increase was slightly but significantly higher in the LIPUS group.

To further confirm these findings, Ki67 staining of sections from these cultures was performed because it has been shown to be a very reliable proliferation marker. The Ki67 index in the LIPUS group, also significantly higher in comparison with the control group, again indicated that LIPUS promotes the proliferation of chondrocytes slightly but significantly. In terms of cartilage regeneration, even a slight increase in the number of chondrocytes is very important. In a previous study performed *in vivo *by Cook and colleagues, cartilage defects in New Zealand rabbits were artificially induced by drilling holes. These defects treated by LIPUS regenerated articular cartilage earlier than the control group, with a hint of increased numbers of chondrocytes [21]. In many previous *in vitro *studies, the cartilage of small animals such as mice and rats has been used. Chondrocytes in cartilage of these animals have a tendency to proliferate more easily, and therefore the regeneration of cartilage is easier than in higher animals. The current study utilized porcine cartilage on the assumption that this is a more appropriate animal model system for the development of future treatments in human cartilage.

### LIPUS promotes production of collagen type-IX

The immunoblotting analysis in the present study indicated that LIPUS increases the production of collagen type-IX, but not collagen type-II. These results suggest that LIPUS transduces the signals through the intracellular signaling pathway that transactivates the collagen type-IX gene. Although the major constituent of the cartilage matrix is type-II collagen, this matrix also contains collagen types of smaller molecular weights, including type VI, type-IX, type X, type XI, and type XII. These collagens all play regulatory roles in maintaining cartilage. Type-IX collagen is present in zones 1 and 2, and it is said to be involved in promoting chondrocyte proliferation and in the expansion of the cartilage layer [22].

In addition, Eyre and colleagues have earlier reported that type-IX collagen accounts for at least 10% of the collagenous protein in fetal cartilage, but only about 1% to 2% of adult hyaline cartilage – and that the ratio of type-IX collagen to type-II collagen decreases as the cartilage matures [23].

Jarmo and coworkers reported that type-IX collagen has unique cell adhesion properties in comparison with other collagen types, and that it provides a novel mechanism for cell adhesion to the cartilaginous matrix [24]. They demonstrated that the type-IX collagen is a superior cell adhesion protein for chondrocytes. In addition to these reports, Wu and colleagues and Blaschke and colleagues suggested that type-IX collagen may be an important stabilizing factor for cartilage type-II collagen fibrils, since it determines the resistance of the fibrils to swelling in the framework of cartilage [25,26]. Hu and colleagues have also reported that type-IX collagen-deficient mice are prone to developing osteoarthritis [27].

The present results suggest that the chondrocyte proliferation in response to LIPUS is associated with the increase in collagen type-IX expression. Eyre and colleagues reported that the ratio of collagen type-IX to collagen type-II in immature cartilage tissue is greater than that in mature cartilage tissue [23]. The results of the current study support their findings. It is likely that the production of collagen type-IX increases in the current system because of an increase in the number of immature chondrocytes in the cultures. In immature chondrocytes, it was reported that the construction of a peripheral matrix with collagen type-IX also promotes the attachment between the cells and the matrix [26].

### Activation of the PI3K/Akt pathway but not the MEK/MAPK pathway by LIPUS

It is very probable that LIPUS transmits signals into the cell via an integrin that acts as a mechanoreceptor on the cell membrane. When ultrasound is transmitted to integrin molecules, this promotes the attachment of various focal adhesion adaptor proteins. Both FAK and Paxillin are in turn phosphorylated as a result of LIPUS exposure initiating this signal transduction.

The integrin/Ras/MAPK/nucleus pathway is considered a general pathway involved in cell proliferation. In the current study, however, MAPK was shown to be similarly activated and phosphorylated regardless of the LIPUS exposure. The results confirmed that MAPK is constitutively activated in both LIPUS-stimulated cells and control cells, probably due to the culture conditions in which the medium is supplemented with 10% FBS. This observation suggests that the significant increase in cell numbers observed in relation to the elevation of type-IX collagen expression is attributable to a signal transduction pathway other than the Ras/MAPK pathway.

The PI3K/Akt pathway, on the other hand, is known to be involved in various functions such as cell survival, proliferation, motility, control of cell size and metabolism [28,29]. In the present experiments, this pathway was found to be newly activated by LIPUS. A previous report also showed that phosphorylated Akt inhibits glycogen synthase kinase-3, which otherwise phosphorylates β-catenin [30]. A high intracellular concentration of β-catenin therefore accumulates when glycogen synthase kinase-3 is inhibited by phosphorylated Akt. In turn, β-catenin translocates to the nucleus and promotes the transcription of its target genes.

**Figure 7:**
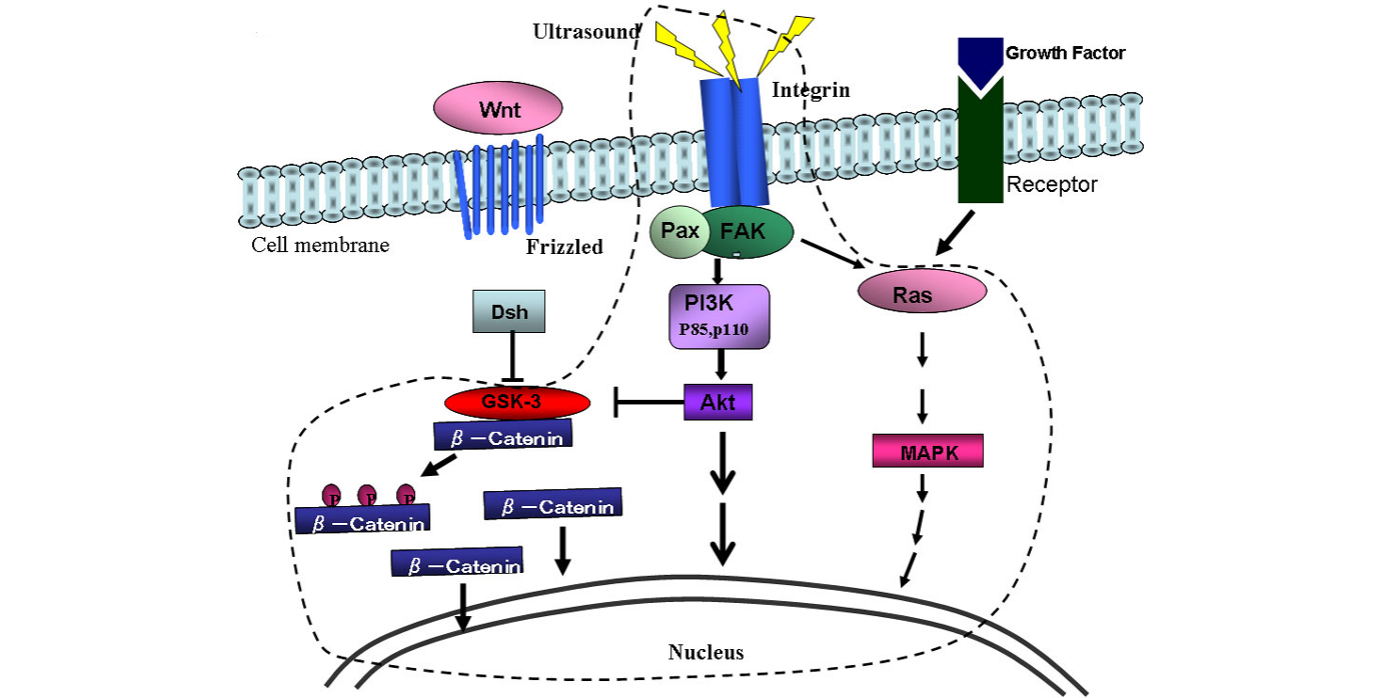
**Signal transduction pathways activated by low-intensity pulsed ultrasound**. Area enclosed with a black broken line is the signaling pathway specified in the present experiment. One of the receptors of low-intensity pulsed ultrasound (LIPUS) is through integrin, and the integrin/mitogen-activated protein kinase (MAPK) pathway is activated to the same extent in both the LIPUS group and the control group. The integrin/phosphatidylinositol 3 kinase (PI3K)/Akt pathway, however, was further activated by LIPUS. The expression of β-catenin, which is downstream of the Akt signaling pathways, is also increased by LIPUS. FAK, focal adhesion kinase; GSK-3, glycogen synthase kinase-3; Pax, Paxillin.

The Wnt signaling pathway may also be involved in the increase in the intracellular β-catenin levels [31]. In the current study, LIPUS was found to significantly increase the number of β-catenin-positive cells during enhanced cell proliferation. Both the PI3K/Akt pathway and the Akt/β-catenin pathway are therefore strongly implicated in this process (Figure 7). Moreover, the expression of the cyclin B_1 _and cyclin D_1 _was found to be elevated in the LIPUS group, providing further evidence that LIPUS promotes the active division of chondrocytes [32,33]. In this regard, Li and colleagues have demonstrated that transforming growth factor beat stimulates cyclin D_1 _expression in chondrocytes in part through the activation of β-catenin signaling [34].

Wnt/β-catenin signaling has been reported to play a crucial role in cell proliferation and in the morphogenesis of chondrocytes [35]. Since there is some functional interaction between the PI3K/Akt pathway and Wnt/β-catenin signaling, LIPUS may activate β-catenin signaling via the PI3K/Akt pathway. As indicated in Figure 4c,d, the nuclear localization of β-catenin, as a marker of the β-catenin signaling, was more prominent in LIPUS-stimulated cells than in the control cells, thus indicating this to be the case.

## Conclusion

LIPUS promotes type-IX collagen accumulation and enhances the proliferation of cultured chondrocytes. In addition to the general growth factor signaling via the Ras/MAPK pathway, mechanical signal transduction to the nucleus through the integrin/PI3K/Akt pathway is activated by LIPUS, thus resulting in an increased matrix production and proliferation of chondrocytes. Akt seems to control the metabolism of β-catenin via glycogen synthase kinase-3, which phosphorylates β-catenin, and also raises the intracellular β-catenin concentration, which in turn promotes its translocation to the nucleus.

In future studies it will be necessary to elucidate the signals or transcription factors that operate downstream of Akt in this system. Certain membrane receptors or ion channels other than integrins, which may reside upstream of the transcription factors that promote the production of collagen type-IX, should also be investigated.

## Abbreviations

3D = three-dimensional; DMEM = Dulbecco's modified Eagle's medium; FAK = focal adhesion kinase; FBS = fetal bovine serum; LIPUS = low-intensity pulsed ultrasound; MAPK= mitogen-activated protein kinase; PBS = phosphate-buffered saline; PCNA = proliferating cell nuclear antigen; PI3K= phosphatidylinositol 3-OH kinase.

## Competing interests

The authors declare that they have no competing interests.

## Authors' contributions

RT performed planning of this study, the *in vitro *experiment, and generalization. AR performed the immunohistochemistry. NK performed western blotting analysis. YM-T was a senior advisor. AF performed cell counting and histological examinations. YT performed western blotting analysis. TS performed ultrasound stimulation. SM was a senior advisor. YY performed histological examinations. KK performed planning and cell culture. IA was a senior advisor. TS was a senior advisor. All authors participated in the conception and design of the study. All authors read and approved the final manuscript.

## References

[B1] Iwata H (1993). Phamacologic and clinical aspects of intraarticular injection of hyaluronate. Clin Orthop.

[B2] Alarid ET, Schlechter NL, Russell SM, Nicoll CS (1992). Evidence suggesting that insulin-like growth factor-I is necessary for the trophic effects of insulin on cartilage growth *in vivo*. Endocrinology.

[B3] Schlechter NL, Russell SM, Spencer EM, Nicoll CS (1986). Evidence suggesting that the direct growth-promoting effect of growth hormone on cartilage *in vivo* is mediated by local production of somatomedin. Proc Natl Acad Sci USA.

[B4] Salter MD, Millward-Sadler JS, Nuki G, Wright OM (2001). Integrin–interleukin-4 mechanotransduction pathways in human chondrocytes. Clin Orthop.

[B5] Sah RL, Kim YJ, Doong JY, Grodzinsky AJ, Plaas AH, Sandy JD (1989). Biosynthetic response of cartilage explants to dynamic compression. J Orthop Res.

[B6] Sah RL, Trippel SB, Grodzinsky AJ (1996). Differential effects of serum, IGF-I, and FGF-2 on the maintenance of cartilage physical properties during long-term culture. J Orthop Res.

[B7] Hangody L, Kish G, Kárpáti Z, Szerb I, Udvarhelyi I (1997). Arthroscopic autogenous osteochondral mosaicplasty for the treatment of femoral condylar articular defects. A preliminary report. Knee Surg Sports Traumatol Arthrosc.

[B8] Ochi M, Uchio Y, Tobita M, Kuriwaka M (2001). Current concepts in tissue engineering technique for repair of cartilage defect. Artif Organs.

[B9] Ochi M, Uchio Y, Matsusaki M, Wakitani S, Sumen Y, Chan KM, FU F (1998). Cartilage repair – a new surgical procedure of cultured chondrocyte transplantation. Controversies in Orthopaedic Sports Medicine.

[B10] Mizuno S, Tateishi T, Ushida T, Glowacki J (2002). Hydrostatic fluid pressure enhances matrix synthesis and accumulation by bovine chondrocytes in 3-D culture. J Cell Physiol.

[B11] Wright M, Jobanputra P, Bavington C, Salter DM, Nuki G (1996). Effects of intermittent pressure-induced strain on the electrophysiology of cultured human chondrocytes: evidence for the presence of stretch-activated membrane ion channels. Clin Sci.

[B12] Takeuchi R, Saito T, Ishikawa H, Takigami H, Dezawa M, Ide C, Itokazu Y, Ikeda M, Shiraishi T, Morishita S (2006). Effects of vibration and hyaluronic acid on activation of 3-D cultured chondrocytes. Arthritis Rheum.

[B13] Nishikori T, Ochi M, Uchio Y, Maniwa S, Kataoka H, Kawasaki K, Katsube K, Kuriwaka M (2002). Effects of low-intensity pulsed ultrasound on proliferation and chondroitin sulfate synthesis of cultured chondrocytes embedded in Atelocollagen gel. J Biomed Mater Res.

[B14] Parvizi J, Parpura V, Greenleaf JF, Bolander ME (2002). Calcium signaling is required for ultrasound-stimulated aggrecan synthesis by rat chondrocytes. J Orthop Res.

[B15] Zhang ZJ, Huckle J, Francomano CA, Spencer RG (2002). The effects of pulsed low intensity ultrasound on chondrocyte viability, proliferation, gene expression and matrix production. Ultrasound Med Biol.

[B16] Huang MH, Ding HJ, Chai CY, Huang YF, Yang RC (1997). Effects of sonication on articular cartilage in experimental osteoarthritis. J Rheumatol.

[B17] Tang CH, Yang RS, Huang TH, Lu DY, Chuang WJ, Huang TF, Fu WM (2006). Ultrasound stimulates cyclooxygenase-2 expression and increase bone formation through integrin, focal adhesion kinase, phosphatidylinositol 3-kinase, and Akt pathway in osteoblast. Mol Pharmacol.

[B18] Kronenberg MH (2003). Developmental regulation of the growth plate. Nature.

[B19] Itoh H, Aso Y, Furuse M, Noishiki Y, Miyata T (2001). A honeycomb collagen carrier for cell culture as a tissue engineering scaffold. Artif Organs.

[B20] Nawa G, Ueda T, Mori S, Yoshikawa H, Fukuda H, Ishiguro S, Funai H, Uchida A (1996). Prognostic significance of Ki 67 (MIB1) proliferation index and p53 over-expression in chondrosarcomas. Int J Cancer.

[B21] Cook DS, Salkeld SL, Popich-Parton LS, Ryaby JP, Jones DG, Barrack RL (2001). Improved cartilage repair after treatment with low-intensity pulsed ultrasound. Clin Orthop.

[B22] Cay MK, Alvin PLK, David FH, Holmes SLS, Michael EG (1985). Type X collagen, a product of hypertrophic chondrocytes. Biochem J.

[B23] Eyre DR, Apon S, Wu JJ, Ericsson LH, Walsh KA (1987). Collagen type IX: evidence for covalent linkages to type II collagen in cartilage. FEBS Lett.

[B24] Jarmo K, Juha J, Mira T, Joni Y, Liosa N, Tiina V, Piia V, Varpu M, Petri N (2004). The fibril-associated collagen IX provides a novel mechanism for cell adhesion to cartilaginous matrix. J Biol Chem.

[B25] Wu JJ, Woods PE, Eyre DR (1992). Identification of cross-linking sites in bovine cartilage type IX collagen reveals an antiparallel type II – type-IX molecular relationship and type IX to type IX bonding. J Biol Chem.

[B26] Blaschke UK, Eikenberry EF, Hulmes DJS, Galla HJ, Bruckner P (2000). Collagen IX nucleates self-assembly and limits lateral growth of cartilage fibrils. J Biol Chem.

[B27] Hu K, Xu L, Cao L, Flahiff M, Brussiau J, Ho K, Setton A, Youn I, Guilak F, Olsen BR, Li Y (2006). Pathogenesis of osteoarthritis-like changes in the joints of mice deficient in type IX collagen. Arthritis Rheum.

[B28] Downward J (2004). PI(3)Kinase, Akt and cell survival. Semin Cell Dev Biol.

[B29] Gustin AJ, Korgaonkar KC, Pincheira R, Li Q, Donner BD (2006). Akt regulates basal and induced processing NF-kB2 (p100) to p 52. J Biol Chem.

[B30] Darren AEC, Dario RA, Philip C, Mirjana A, Brian AH (1995). Inhibition of glycogen synthase kinase-3 by insulin mediated by protein kinase B. Nature.

[B31] Shtutman M, Zhurinsky J, Simcha I, Albanese C, Pestell MR, Ben-Ze' A (1999). The cyclin D_1 _gene is a target of the β-catenin/LEF-1 pathway. Cell Biol.

[B32] Hwang A, McKenna WG, Muschel RJ (1998). Cell cycle-dependent usage of transcriptional start sites. A novel mechanism for regulation of cyclin B1. J Biol Chem.

[B33] Sgerr CJ (1996). Cancer cell cycles. Science.

[B34] Li TF, Chen D, Wu Q, Chen M, Sheu TJ, Schwarz EM, Drissi H, Zuscik M, O'Keefe RJ (2006). Transforming growth factor-β stimulates cyclin D_1 _expression through activation of β-catenin signaling in chondrocytes. J Biol Chem.

[B35] Yano F, Kugimiya F, Ohba S, Ikeda T, Chikuda H, Ogasawara T, Ogata N, Takato T, Nakamura K, Kawaguchi H, Chung U (2005). The canonical Wnt signaling pathway promotes chondrocyte differentiation in a Sox9-dependent manner. Biochem Biophys Res Commun.

